# Association Between Oral Cavity Cancer and Lymphoma: A Bidirectional Mendelian Randomization Analysis

**DOI:** 10.3290/j.ohpd.c_2780

**Published:** 2026-08-26

**Authors:** Yayan Deng, Min Kang

**Affiliations:** a Yayan Deng Laboratory Technologist, Department of Radiation Oncology, The First Affiliated Hospital of Guangxi Medical University, Nanning, Guangxi, China. Clinical Laboratory Center, The First Affiliated Hospital of Guangxi Medical University, Nanning, Guangxi, China. Conceptualization, formal analysis, methodology, visualization, wrote the original draft.; b Min Kang Professor, Department of Radiation Oncology, The First Affiliated Hospital of Guangxi Medical University, Nanning, Guangxi, China; Guangxi Key Laboratory of Immunology and Metabolism for Liver Diseases, Nanning, Guangxi, China; Key Laboratory of Early Prevention and Treatment for Regional High Frequency Tumour, Ministry of Education, Nanning, Guangxi, China. Conceptualization, runding acquisition, reviewed and edited the manuscript.

**Keywords:** causal relationship, lymphoma, Mendelian randomization, oral cavity cancer.

## Abstract

**Purpose:**

Emerging evidence suggests a potential association between oral cavity cancer (OCC) and lymphoma. This study aimed to investigate whether this association reflects a causal relationship.

**Materials and Methods:**

A two-sample bidirectional Mendelian randomization (MR) approach was utilized to screen genetic instrumental variables (IVs) obtained from genome-wide association studies (GWAS) of OCC and lymphoma, applied various MR methods (inverse variance weighted [IVW] as the main method) to assess the causal relationship between the two malignancies, and performed sensitivity, heterogeneity, and pleiotropic tests to evaluate reliability.

**Results:**

No bidirectional causal association between OCC and Hodgkin lymphoma (HL) or overall non-Hodgkin lymphoma (NHL) was found. Subgroup analysis also showed that no noteworthy bidirectional causal relationship between OCC and NHL subtypes. However, we identified a statistically significant inverse correlation between mature T/NK-cell lymphoma and OCC (IVW: odds ratio [OR] 0.985, 95% confidence interval [CI]: 0.820-0.978, p=0.013). Patients initially diagnosed with mature T/NK-cell lymphoma would have a decreased risk of OCC.

**Conclusion:**

This MR study ruled out the causal relationship between OCC and lymphoma. Future multiethnic studies with substantial sample sizes are need to replicate our findings.

Oral cavity cancer (OCC) is a common head and neck cancer (HNC). OCC develops from the mucosal epithelium in the oral cavity and includes buccal mucosa, the mobile tongue, floor of the mouth, anterior tongue, alveolar ridges, retromolar trigone, and hard palate.^25^ Cancers of the lip and oral cavity are the sixteenth cancer worldwide, with 389,846 new cases and 188,438 deaths in 2022.^20^ With the deepening exploration of OCC, accumulating evidence has revealed that oral microbiome dysbiosis and chronic inflammation play pivotal roles in the initiation and progression of OCC, offering new avenues for predicting patient prognosis and therapeutic response.^1,13,24,54,58,59,65^ Meanwhile, continual advances in diagnostic and therapeutic technologies—particularly the development of bioinformatics approaches such as machine learning and artificial intelligence, along with the clinical application of liquid biopsy—have provided critical support for early detection, precise diagnosis, and treatment monitoring of OCC, thereby markedly improving patient survival rates.^30,51^ With increasing life expectancy, OCC survivors face a heightened risk of developing second primary cancers (SPCs), either concurrently or subsequently, adversely impacting survival outcomes.^32,45^ The standardized incidence ratio of developing SPCs was 1.47-2.8 among patients with OCC in different regions.^15,39,40^ The risk of SPCs was higher among patients initially diagnosed with OCC compared to those with other malignancies in Switzerland.^19^ In Taiwan, the SPCs rates for OCC was 15.7%, with an annual increase rate of 2.9%. This trend led to significantly lower 5-year overall survival (OS) compared to patients without SPCs.^34^ Lymphoma is one of the SPCs following a primary OCC.^39^ Lymphoma originates in lymph nodes and other lymphoid tissues, and is traditionally divided into Hodgkin lymphoma (HL) and non-Hodgkin lymphoma (NHL).^4,12^ Interestingly, among patients with an initial tumor of lymphoma, the risk of OCC as a SPC increased.^5,11^ Moreover, OCC and NHL reciprocally manifested as both the first primary cancer (FPC) and SPC.64 Previous observational studies have indicated a potential interaction between OCC and lymphoma. However, due to the inherent limitations of observational studies, such as confounding factors, whether there exists a causal relationship between OCC and lymphoma remains uncertain.

Unlike observational studies, Mendelian randomization (MR) is a statistical methodology that employs single-nucleotide polymorphisms (SNPs) as instrumental variables (IVs) to infer the causal relationship between exposure and outcome.^47^ MR is grounded in the principle of the random allocation of alleles to avoid the impact of confounding factors.^14^ In recent years, numerous studies have employed MR methods to infer pathogenic associations between FPC and SPC. Ruan et al^49^ conducted a pan-cancer analysis and MR study to support the association between a certain type of FPC and SPC. Similarly, bidirectional MR analysis provided evidence for an elevated risk of second primary colorectal cancer (CRC) following primary lung cancer, as well as a causal effect of breast cancer (BC) on the development of second primary lung adenocarcinoma.^33,60^ Contrary to the aforementioned findings, Liu et al^36^ ruled out the association between primary BC and CRC at the genetic level using a two-sample MR analysis.

The present study hypothesized that OCC and lymphoma may be causally related rather than merely correlated due to shared risk factors or reverse causation. Using summary statistics from large-scale genome-wide association studies (GWAS) of OCC and lymphoma, a bidirectional Mendelian randomization analysis was conducted to test two directional hypotheses: (i) a genetically proxied liability to OCC causally influences the risk of lymphoma, and (ii) a genetically proxied liability to lymphoma causally influences the risk of OCC. Establishing such causal direction could motivate mechanistic investigations into potentially shared pathways underlying these two malignancies and provide evidence to inform clinical practice. Conversely, if neither direction is supported, the observed association between the two malignancies is more likely attributable to shared risk factors or confounding related to treatment and/or environmental exposures.

## MATERIALS AND METHODS

### Study Design

**Fig 1:**
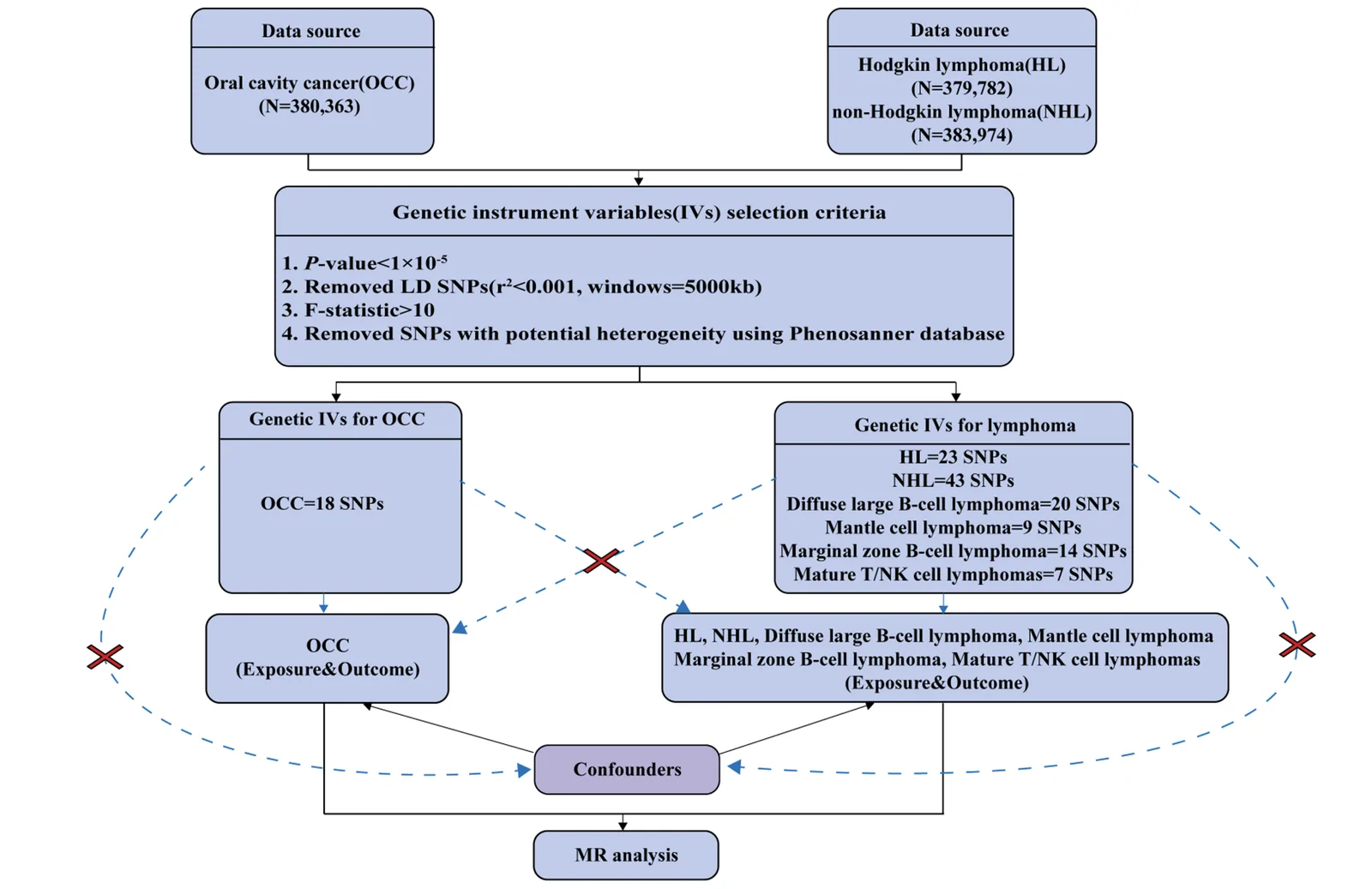
Study design and overview of the Mendelian randomization (MR) study.

Through the two-sample bidirectional MR analysis, the causal relationship between OCC and lymphoma was explored. The overall study design is shown in Fig 1. In the MR analysis, SNPs used as IVs should satisfy the following three assumptions: (1) IVs are strongly correlated with the exposure; (2) there is no correlation between IVs and confounding factors of outcomes; (3) IVs affects outcomes only through exposure.^17,50^

### Data Source

GWAS summary statistics for OCC, HL and NHL were acquired from the FinnGen research project, with all participants being of European descent.^31^ The OCC GWAS data included 1614 cases and 378,749 controls. Regarding the reverse analysis, we employed genetic IVs associated with HL and overall NHL, which were derived from HL GWAS data (1,003 cases) and overall NHL GWAS data (5225 cases) respectively. The overall NHL GWAS data included subgroup for diffuse large B-cell lymphoma (1505 cases), mantle cell lymphoma (472 cases), marginal zone B-cell lymphoma (513 cases), and mature T/NK cell lymphoma (440 cases). The details of the GWAS summary statistics used in our study are provided in Supplementary Table S1xxLINK. No specific ethical approval or written informed consent from participants was deemed necessary, because all data are publicly accessible.

### Selection of IVs

To satisfy the three critical assumptions above, genetic IVs for our study were selected according to the following criteria: (1) SNPs reached a genome-wide statistical significance level (p<5.0E-08). In selecting genome-wide SNPs statistically significantly associated with OCC, the limited sample size of SNPs were identified; therefore, we relaxed the p threshold to 1.0E-05; (2) r^2^ measure of linkage disequilibrium (LD) among SNP <0.001 at a clumping window of 5000 kb; (3) The strength of each remaining SNP was measured using the F-statistics by the formula F = R2[(N – 2) / (1 – R2)] (where N refers to the total number of samples and R^2^ indicates the proportion of the variance of the exposure factor explained by the IVs), with a threshold of more than 10; (4) the PhenoScanner database was used to exclude SNPs linked to confounding factors.

### MR Analysis

Six different MR methods were utilized to assess the genetic-level associations between OCC and lymphoma, including two inverse variance weighting (IVW) methods (random effect IVW and fixed effect IVW) and four additional techniques: weighted median (WM), MR-Egger, weighted mode and simple mode. The IVW method was performed for principal analysis. IVW, which employs meta-analysis approach to combine ratio estimates of SNPs in an inverse variance weighted manner, is widely used for MR analysis.^3,6^ The random-effect IVM method was applied where heterogeneity was observed in the MR analysis, while the fixed-effect IVM approach was utilized in the absence of heterogeneity.^41^ However, IVW assumes that all SNPs serve as valid instrumental variables, which requires additional validation through other methods. The WM method was utilized in this study due to its high tolerance for pleiotropic genetic variation, which allows for reliable estimation even when nearly half of the IVs are invalid.^3^ MR-Egger regression method, which performs a linear regression analysis with weighted coefficients for the outcome and exposure variables, can detect certain deviations from the standard IVs assumptions and provides a non-violation-prone effect estimate.^7^ In scenarios involving pleiotropy, the MR-Egger regression technique was employed.

### Sensitivity Analysis

Cochran’s IVW Q statistics were employed to evaluate heterogeneity, and a p-value<0.05 was regarded as indicating statistically significant heterogeneity. MR-Egger regression was utilized to assess potential pleiotropic effects of IVs. The close to-zero intercept in the MR-Egger regression can indicate that the results of MR analysis are not affected by horizontal pleiotropy.^2^ Leave-one-out sensitivity analysis was also performed to assess the presence of single SNP responsible for the causal effect.

Results were presented as odds ratio (OR) with corresponding 95% confidence intervals (CI). The R package (version 4.0.1) “TwoSample MR” was predominantly utilized for conducting statistical analyses in MR.^22,23^ A comprehensive statistical analysis was performed, and statistical significance was determined at p < 0.05.

## RESULTS

### Screen of IVs

After rigorous screening, a total of 18 SNPs were identified as OCC IVs in OCC to lymphoma MR analysis. In the reverse MR analysis, we selected 23 SNPs for HL, 43 SNPs for NHL, 20 SNPs for diffuse large B-cell lymphoma (DLBCL), 9 SNPs for mantle cell lymphoma, 14 SNPs for marginal zone B-cell lymphoma and 7 SNPs for mature T/NK cell lymphoma as IVs. The F-statistics for all exposures were>10, suggesting that IVs were robust.^8^ The details of the SNPs used in the present study are provided in Table S2 xxLINK.

### MR Analysis Results of OCC to Lymphoma

**Fig 2:**
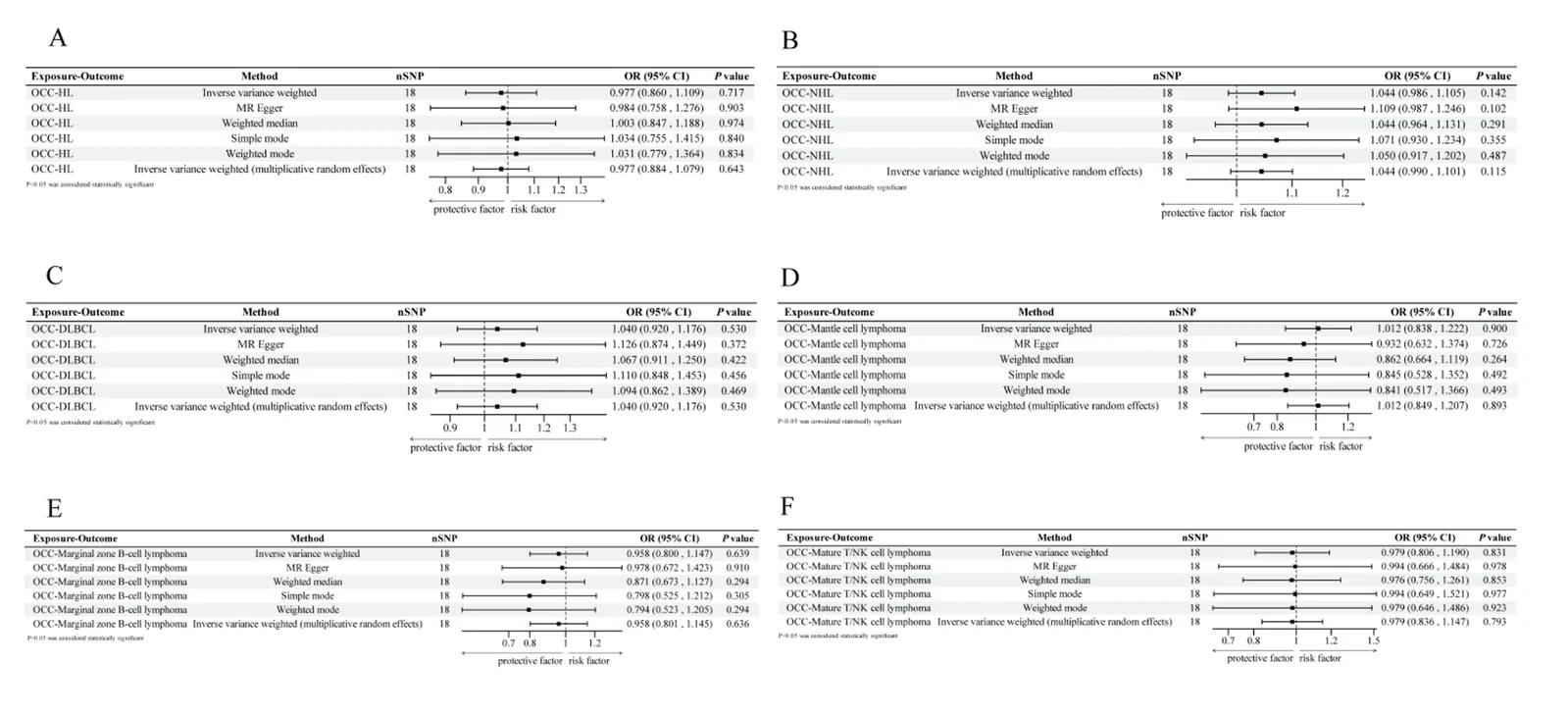
Forest plot of a TWO-SAMPLE Mendelian randomization analysis examining the causal association between OCC and lymphoma. A to F: The MR results of causal effects of OCC on HL (A), overall NHL (B), DLBCL (C), mantle cell lymphoma (D), marginal zone B-cell lymphoma (E), and mature T/NK cell lymphoma (F). HL: Hodgkin lymphoma; NHL: non-Hodgkin lymphoma; DLBCL: diffuse large B-cell lymphoma; IVW: inverse variance weighted; SNP: single nucleotide polymorphism; OR: odds ratio; CI: confidence interval.

**Fig 3:**
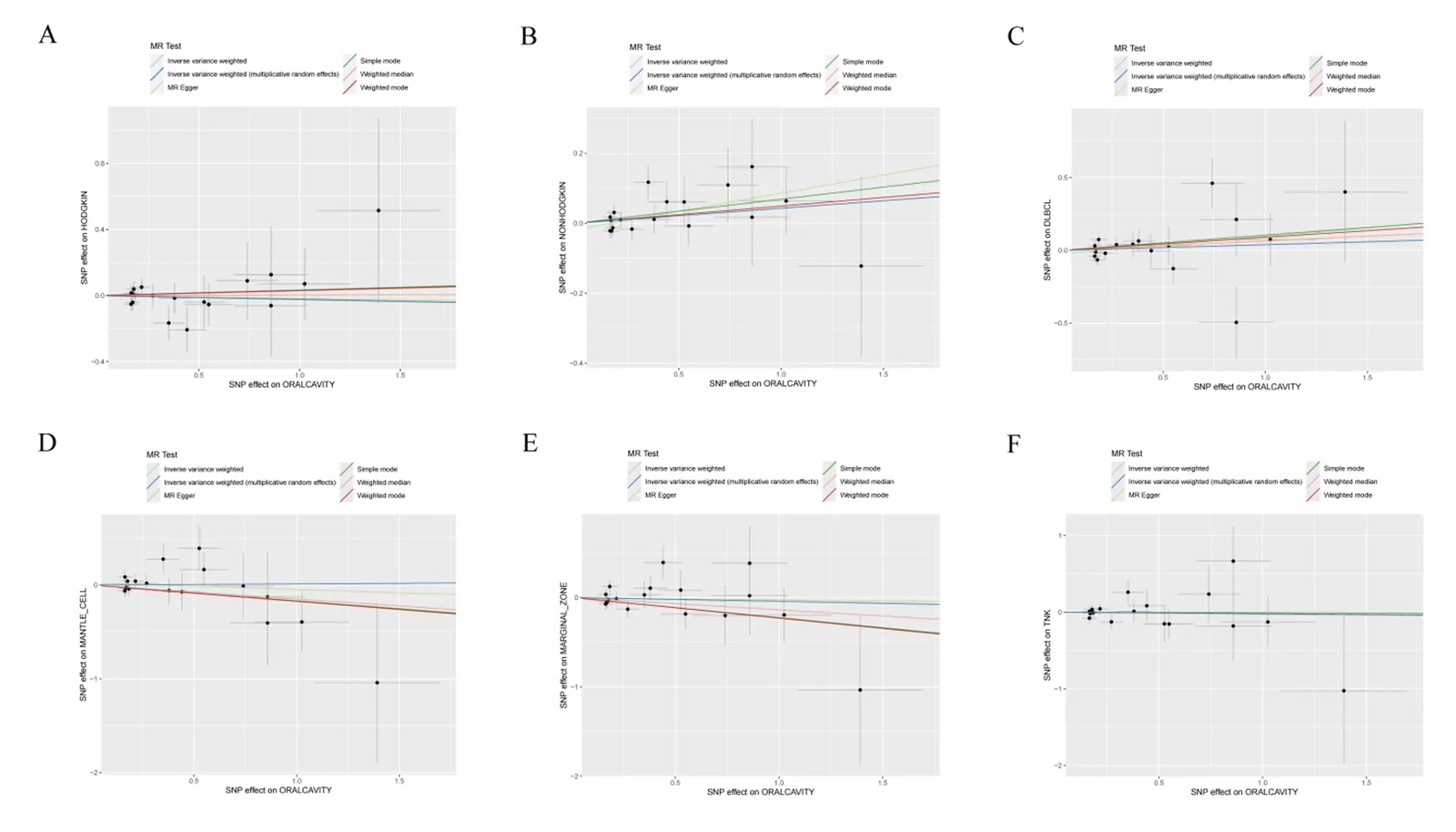
Scatter plots representing genetic instrumental variables association between OCC and lymphoma. A to F: Plots of the effect size of each single nucleotide polymorphism of OCC on HL (A), overall NHL (B), DLBCL (C), mantle cell lymphoma (D), marginal zone B-cell lymphoma (E), and mature T/NK cell lymphoma (F) risk. HL: Hodgkin lymphoma; NHL: non-Hodgkin lymphoma; DLBCL: diffuse large B-cell lymphoma; IVW: inverse variance weighted.

In forward–direction MR analysis, the IVW method indicated the absence of a causal relationship between OCC and either HL or overall NHL (HL: OR=0.977, 95% CI=0.860-1.109, p=0.717; NHL: OR=1.044, 95% CI=0.860-1.109, p=0.717, Figs 2A and 2B). According to the WHO classification scheme, NHL includes different subtypes. The causal effects of OCC on NHL subtypes were further analyzed by the IVW method. Consistently, there were no causal effects of OCC on NHL subtypes (DLBCL: OR=1.040, 95% CI=0.920-1.756, p=0.530; mantle cell lymphoma: OR=1.012, 95% CI=0.0838-1.222, p=0.900; marginal zone B-cell lymphoma: OR=0.958, 95%CI=0.800-1.147, p=0.639; mature T/NK cell lymphoma: OR=0.979, 95% CI=0.806-1.190, p=0.831, Figs 2C to 2F). Additionally, no causal effect of OCC on lymphoma was observed using the other MR methods (WM, MR-Egger, weighted mode and simple mode) (Figs 2 and 3). Thus, there was no genetic evidence to suggest that patients diagnosed with OCC were at an increased risk of second primary lymphoma.

### MR Analysis Results of Lymphoma to OCC

**Fig 4:**
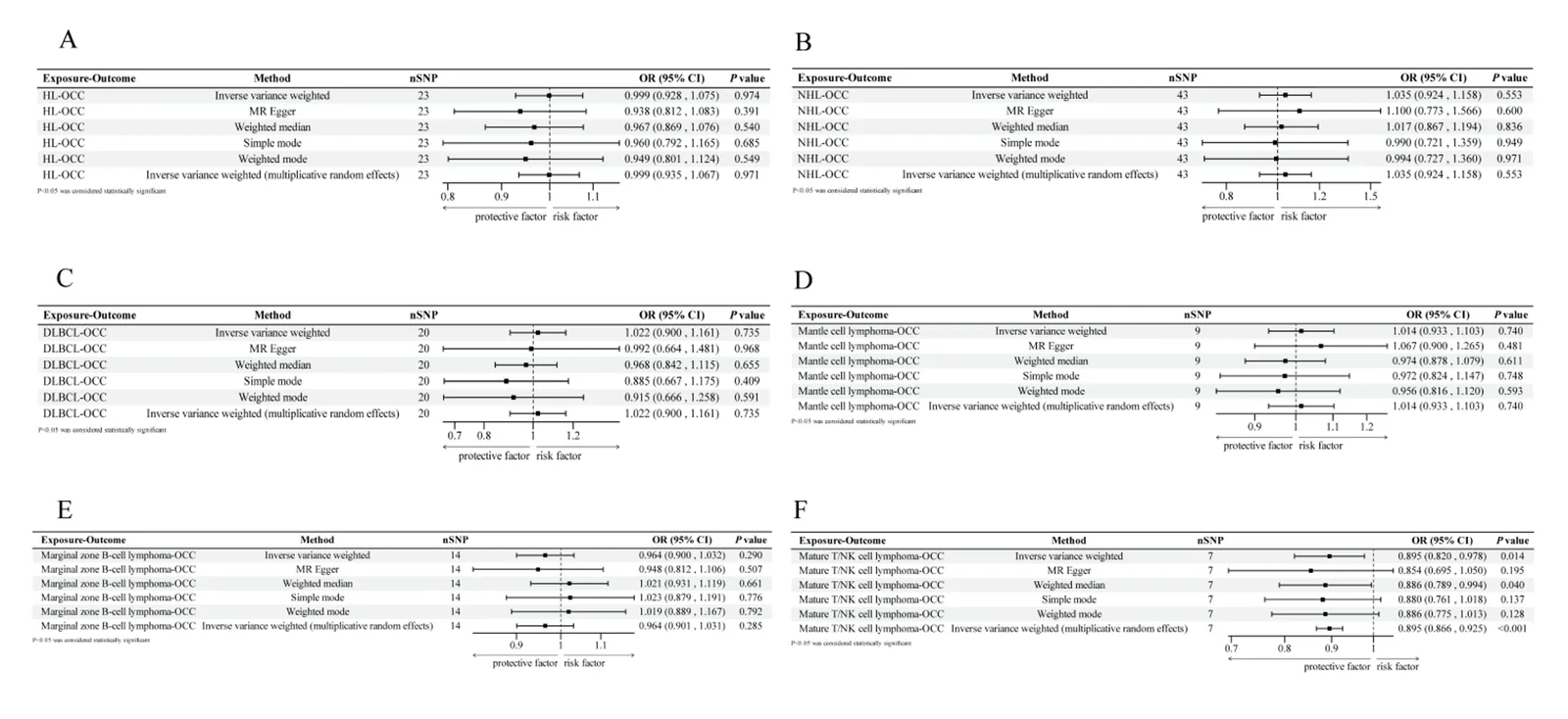
Forest plot of a two-sample Mendelian randomization (MR) analysis examining the causal association between lymphoma and OCC. A to F: MR results of causal effects of HL (A), overall NHL (B), DLBCL (C), mantle cell lymphoma (D), marginal zone B-cell lymphoma (E), and mature T/NK cell lymphoma (F) on OCC. HL: Hodgkin lymphoma; NHL: non-Hodgkin lymphoma; DLBCL: diffuse large B-cell lymphoma; IVW: inverse variance weighted; SNP: single nucleotide polymorphism; OR: odds ratio; CI: confidence interval.

**Fig 5:**
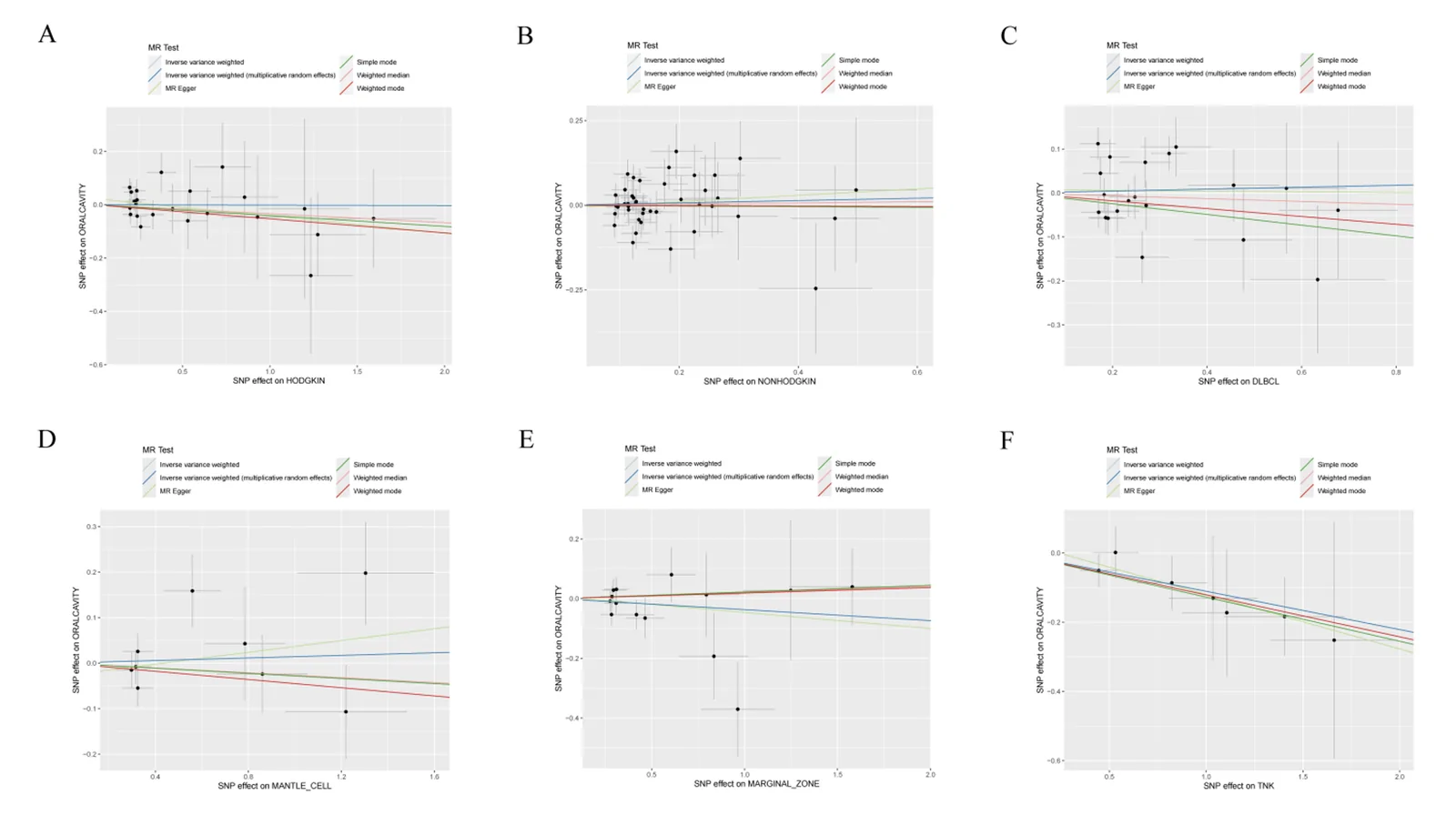
Scatter plots representing genetic instrumental variables association between lymphoma and OCC. A to F: Plots of the effect size of each single nucleotide polymorphism of HL (A), overall NHL (B), DLBCL (C), mantle cell lymphoma (D), marginal zone B-cell lymphoma (E), and mature T/NK cell lymphoma (F) on OCC risk. HL: Hodgkin lymphoma; NHL: non-Hodgkin lymphoma; DLBCL: diffuse large B-cell lymphoma; IVW: inverse variance weighted.

In reverse-direction MR analysis, the causal effects of HL and overall NHL on OCC were analyzed first. No statistically significant causal effects of HL or overall NHL on OCC were observed using the IVW method (HL: OR=0.998, 95% CI=0.928-1.075, p=0.974; NHL: OR=1.035, 95% CI=0.924-1.158, p=0.553, Figs 4A and 4B, Figs 5A and 5B). About stratified analysis, the IVW method and WM method showed inverse correlation between mature T/NK cell lymphoma and OCC (IVW: OR=O.895, 95% CI=0.820-0.978, p=0.013, WM: OR=0.086, 95% CI=0.789-0.994, p=0.039, Figs 4F and 5F), while the absence of statistically significant causal links between other NHL subtypes and OCC (Figs 4C to 4E, Figs 5C to 5E). Taken together, patients with mature T/NK cell lymphoma may have a decreased risk of OCC.

### Horizontal Pleiotropy and Heterogeneity Test

In the MR analysis of DLBCL to OCC, significant heterogeneity was detected among the DLBCL IVs (MR-Egger’s Q value = 39.966, p=0.002; IVW: Cochran’s Q value = 40.020, p=0.003). No heterogeneity was found in any other MR analysis group (Table S3). The MR-Egger regression analysis indicated that there was no horizontal pleiotropy of the IVs in all MR analysis (Fig S1 https://www.quintessence-publishing.com/quintessenz/journals/articles/downloads/ohpd_2026_8120_deng_kang_suppl_fig_1.tif). The leave-one-out analyses indicated that no single SNP exerted a substantial disproportionate influence on the causality estimates (Fig S2 https://www.quintessence-publishing.com/quintessenz/journals/articles/downloads/ohpd_2026_8120_deng_kang_suppl_fig_2.tif). Therefore, the results of the MR analysis showed robustness and reliability.

## DISCUSSION

Advancements in screening programs and therapeutic procedures have facilitated the earlier detection of cancers, and improved treatment approaches have substantially enhanced OS rates in cancer patients. Due to substantial progress in malignancy prevention, diagnosis, and management, the growing and aging population of FPC survivors has contributed to an increased burden of SPC. Multiple factors predispose cancer survivors to develop SPC, including genetic predisposition, environmental exposures, and therapeutic influences.^57^ Over recent decades, SPC has emerged as a significant cause of mortality. Investigating the risk factors for SPC facilitates the identification of high-risk populations, enabling clinicians to implement surveillance and provide timely interventions.

Previous retrospective observational studies have indicated that OCC and lymphoma had bidirectional associations as FPC and SPC.^35,39^ To investigate whether there is a genetic causal relationship between OCC and lymphoma, a two-sample bidirectional MR analysis was performed in this study. Our findings suggest that there is no genetic causal relationship between OCC and HL or overall NHL. Furthermore, the overall NHL was stratified into subtypes (DLBCL, mantle cell lymphoma, marginal zone B-cell lymphoma, mature T/NK cell lymphoma) and examine the genetic causal association between OCC and NHL subtypes. Our analysis revealed no evidence of a causal association between OCC and subtypes of NHL. However, a statistically significant inverse correlation was observed between mature T/NK cell lymphoma and OCC, suggesting patients initially diagnosed with mature T/NK-cell lymphoma would have a decreased risk of OCC. Collectively, the present findings do not support the existence of a causal relationship between OCC and lymphoma.

Contrary to the findings of this study, a recent MR study demonstrated that patients diagnosed with primary oral and pharyngeal cancer exhibited a significantly elevated risk of developing second primary NHL.^49^ The divergence in outcomes may be ascribed to several factors. Primarily, the GWAS summary statistics utilized in the two studies were derived from distinct databases, potentially introducing variability in the data. Secondly, our MR analysis was specifically confined to OCC and lymphoma, deliberately excluding cases of oropharyngeal cancer. Furthermore, the relatively limited number of cases pertaining to oral and pharyngeal cancers in their findings could attenuate the statistical robustness and persuasiveness of the results. The observed inconsistency underscores the imperative for independent replication across additional datasets to corroborate these findings.

Although the causal relationship between OCC and lymphoma requires further substantiation, it is indisputable that a correlation exists between them. Given that radiotherapy, a key treatment for OCC and lymphoma, has been established as a risk factor for SPC, we hypothesize that the association between OCC and lymphoma may be attributable to radiotherapy. Indeed, previous studies have identified an elevated risk of SPC in patients with OCC or lymphoma subsequent to radiotherapy.^9,21,38,46,48,62,66^ For instance, patients with OCC who received radiotherapy alone or in combination with surgery exhibited an elevated risk of developing second primary lymphoma, but not among those who received surgery alone.^21^ Moreover, patients diagnosed with early-stage NHL of the head and neck exhibited a markedly increased risk of developing OCC, after radiotherapy.^56^ Nevertheless, certain studies have suggested that radiotherapy does not elevate the risk of SPC in OCC or lymphoma.^18,42,44^ Consequently, further research is required to examine the impact of radiotherapy modalities, radiation field dimensions, and dosage to elucidate whether radiotherapy facilitates the development of SPC in OCC or lymphoma survivors. In addition to the long-term effects of radiotherapy, other factors, such as smoking, may contribute to the association between OCC and lymphoma. Smoking not only contributes to the pathogenesis of OCC or lymphoma but also augments the risk of SPC among OCC survivors.^10,16,26,52,53,55^ Hence, the role of smoking in the bidirectional relationship between OCC and lymphoma merits further investigation.

In the present study, we conducted a bidirectional MR analysis between OCC and subtypes of NHL, revealing that the presence of mature T/NK cell lymphoma was inversely correlated with the development of OCC. This suggests that the pathogenic mechanisms of these two malignancies may be mutually exclusive in certain aspects. Notably, neuro-oncological ventral antigen 1 (NOVA1) may play a pivotal role in this context. NOVA1, an RNA-binding protein crucial for post-transcriptional regulation, functions as an oncogene in various tumors.^37,43,61,63^ Upregulated NOVA1 expression was specific to mature T/NK cell lymphoma and correlated with shorter OS rate in mature T cell lymphoma.^29^ Conversely, NOVA1 expression was reduced in OCC, where lower NOVA1 level was associated with fewer tumor-infiltrating lymphocytes and worse prognosis, while higher NOVA1 level correlated with increased immune cell infiltration.^27^ Additionally, NOVA1 suppression seemed to be involved in immune cell dysregulation.^28^ Therefore, we hypothesize that high NOVA1 expression in mature T/NK cell lymphoma may suppress the development of second primary OCC by modulating the immune microenvironment essential for oral carcinogenesis. Further analysis of the abundance and functionality of immune cells within the tumor microenvironment of both malignancies could clarify their causal relationship. In addition, other oncogenic mechanisms should also be investigated to gain a more comprehensive understanding of the negative correlation between mature T/NK cell lymphoma and OCC.

In this study, bidirectional MR analysis was employed, a method that mitigates unobserved confounding and reverse causation, to assess the causal relationship between OCC and lymphoma, thereby providing additional evidence for their association. Our findings suggest biologic heterogeneity in the association between OCC and distinct lymphoma subtypes. The reduced risk of OCC among patients with mature T/NK cell lymphoma may be attributable to subtype-specific differences in tumor-immune interactions. These results provide a new perspective for research on both diseases and for optimizing therapeutic strategies, highlighting that clinicians should focus on their distinct pathogenic mechanisms and develop more targeted precision-treatment approaches. In addition, our study may inform refinement of risk stratification and surveillance strategies in lymphoma, underscoring that subtype differences should be explicitly considered in OCC risk assessment and follow-up management among lymphoma patients. However, this study has several limitations. The GWAS summary statistics were exclusively derived from individuals of European descent, and the restricted sample size limits the generalizability of the results to diverse ethnic populations. Further validation through large-scale, multi-ethnic studies is warranted. Moreover, MR analysis may only partially reflect the biological mechanisms underlying the causal relationship between exposures and outcomes, and conclusive causal inferences require additional validation via biological mechanistic investigations.

## CONCLUSION

Our findings indicated that there was no increased risk of developing SPC-lymphoma after OCC or SPC-OCC following lymphoma at the genetic level. Future multiethnic investigations encompassing substantial sample sizes are imperative to replicate our findings.

## ACKNOWLEDGEMENTS

We would like to express our gratitude to the FinnGen database for providing publicly accessible data on oral cavity cancer and lymphoma. This work was supported by the National Natural Science Foundation of China (grant number 82272736).

## AI Statement

Artificial intelligence (AI) tools were used for language polishing and refinement of English expression during manuscript preparation.
